# Cytotoxicity, Oxidative Stress, Cell Cycle Arrest, and Mitochondrial Apoptosis after Combined Treatment of Hepatocarcinoma Cells with Maleic Anhydride Derivatives and Quercetin

**DOI:** 10.1155/2017/2734976

**Published:** 2017-10-10

**Authors:** Gabriela Carrasco-Torres, Rafael Baltiérrez-Hoyos, Erik Andrade-Jorge, Saúl Villa-Treviño, José Guadalupe Trujillo-Ferrara, Verónica Rocío Vásquez-Garzón

**Affiliations:** ^1^Departamento de Bioquímica y Sección de Graduados, Escuela Superior de Medicina del IPN, Ciudad de México, Mexico; ^2^CONACYT, Facultad de Medicina y Cirugía, Universidad Autónoma Benito Juárez de Oaxaca, Oaxaca de Juárez, OAX, Mexico; ^3^Departamento de Biología Celular, Centro de Investigación y de Estudios Avanzados del Instituto Politécnico Nacional, Ciudad de México, Mexico

## Abstract

The inflammatory condition of malignant tumors continually exposes cancer cells to reactive oxygen species, an oxidizing condition that leads to the activation of the antioxidant defense system. A similar activation occurs with glutathione production. This oxidant condition enables tumor cells to maintain the energy required for growth, proliferation, and evasion of cell death. The objective of the present study was to determine the effect on hepatocellular carcinoma cells of a combination treatment with maleic anhydride derivatives (prooxidants) and quercetin (an antioxidant). The results show that the combination of a prooxidant/antioxidant had a cytotoxic effect on HuH7 and HepG2 liver cancer cells, but not on either of two normal human epithelial cell lines or on primary hepatocytes. The combination treatment triggered apoptosis in hepatocellular carcinoma cells by activating the intrinsic pathway and causing S phase arrest during cell cycle progression. There is also clear evidence of a modification in cytoskeletal actin and nucleus morphology at 24 and 48 h posttreatment. Thus, the current data suggest that the combination of two anticarcinogenic drugs, a prooxidant followed by an antioxidant, can be further explored for antitumor potential as a new treatment strategy.

## 1. Introduction

The increase in the growth, proliferation, and survival of cancer cells is due to genetic and epigenetic changes that result in the modification of hundreds of genes that finally induce aberrations in multiple pathways. One of these alterations includes the reprogramming of metabolism due to the requirement of high levels of energy, nucleotides, amino acids, and lipids for rapid cell growth and proliferation [1]. The increased requirement for ATP by mitochondrial oxidative phosphorylation generates free oxygen radicals that induce oxidative stress, and under hypoxic or anoxic conditions, cancer cells resolve their energy demand by utilizing glucose as a source of energy [2, 3]. Metabolic adaptations are critical for the capability of cancer cells to sustain proliferation. Reactive oxygen species (ROS) are produced due to the increase in metabolic activity and due to the activation of oncogenes and functional loss of p53. To modulate the disturbance in redox balance during the process of carcinogenesis, cancer cells increase antioxidant defenses and upregulate prosurvival molecules [4, 5]. Cancer cells exhibit enhanced intracellular levels of glutathione (GSH) and gamma-glutamylcysteine synthetase and activate the transcription factors NfkB, HIF, p53, and FoxM1 [5, 6]. GSH is one of the principal antioxidants involved in many cellular processes. Nrf2, an oncogenic transcription factor, regulates intracellular stress and plays a key role in the environmental control of the abundant cellular antioxidant systems responsible for GSH production [7]. The modulation of antioxidative defense systems allows tumor cells to bypass cell death caused by excessive levels of ROS. However, excessive ROS production can affect cancer cells, resulting in cell cycle arrest and apoptosis [8].

Chemotherapy is considered a promising way of treating cancer. In addition, selective targeting of cancer cells by the modulation of ROS production has been proposed as an excellent therapeutic alternative. Chemotherapeutic drugs such as amino benzenesulfonamide induce apoptosis, increase ROS, and reduce GSH levels [8]. Novel drugs have been identified, which increase ROS levels and modulate the mitochondrial membrane potential, making tumor cells susceptible to cell death. Many reports have indicated that antitumor agents exert their effects by inducing ROS, but the exact mechanism of ROS generation is not known [9]. Cancer-related multidrug resistance is associated with elevated GSH levels [10]. One of the principal criteria for potential anticancer drugs is the maximum effect on cancer cells with minimum damage to adjacent normal cells. Additionally, in recent years, there has been an increase in the demand for the development of new and effective antitumor drugs at affordable prices. The use of antitumor compounds with oxidative capacity does not harm normal cells because these drugs amplify the levels of reactive oxygen species, but the production of ROS in normal cells is regulated efficiently by the antioxidant defense system. The production of endogenous ROS in cells is regulated by enzymatic reactions mainly in the mitochondria. Flavonoids have emerged as alternative cancer treatment agents because of their multiple mechanisms of action and limited toxicity. Some flavonoids have antioxidant properties, and some induce oxidative stress, but flavonoids are less toxic than conventional therapies [10].

Quercetin is one of the most abundant flavonoids found in vegetables and fruits [11]. The cancer preventive mechanisms of quercetin include antioxidation and promotion of cell cycle arrest and cell death [12]. The anticancer effect of quercetin is mediated through their free radical-scavenging activity. Quercetin has been found to induce apoptosis via the inhibition of the Akt-CSN6-Myc signaling axis in colon cancer cells [13]. Likewise, the anticarcinogenic action of quercetin has been observed to be mediated by the downregulation of phosphatidylinositol 3-kinase (PI3K) and protein kinase C (PKC) via the induction of p53 in hepatocellular carcinoma [14]. It has been reported that quercetin delivered in the form of nanoparticles induces ROS production and p53 loss, arrests the cell cycle in the sub-G phase, and induces apoptosis by mitochondrial pathways in HepG2 cells [15]. Despite the various mechanisms of quercetin in eliminating tumor cells and its numerous effects, several studies have reported that it does not harm normal cells [16, 17]. Nevertheless, contradicting reports exist regarding the exact mechanism of action of quercetin. However, *in vitro* and *in vivo* studies have shown that quercetin potentiates the anticancer effect of some anticancer drugs, and in addition to being chemically synthesized and commercially sold, it has the advantage of being a component of diet [11].

Quercetin is effective against multiple targets involved in cancer development and progression. The goal of this study was to combine quercetin with maleic anhydride derivatives to enhance their ability to selectively kill tumor cells but not normal cells. Maleic anhydride derivatives have anticancer effects, and they are strong prooxidant compounds with a preference for cysteine [18].

## 2. Materials and Methods

### 2.1. Synthesis of Maleic Anhydride Derivatives

Synthesis was performed according to the method described in Trujillo-Ferrara et al. (1994) [19]. Briefly, 0.050 moles of maleic anhydride was dissolved in 75 mL of tetrahydrofuran at a 1 : 2 molar ratio. The exothermic reaction was maintained under vigorous stirring at room temperature for 60 minutes. The compound was separated by filtration and washed with cold ethanol (4°C), followed by incubation at 40°C in a vacuum oven for drying. The 3′5′-dimaleimylbenzoic acid product was obtained in 98% yield. Next, maleimide was obtained by cyclization of its 3,5-dimaleamylbenzoic acid intermediate precursor through the dehydration of the maleamide group by mixing 0.028 moles of 3,5-dimaleamylbenzoic acid with 0.056 moles of anhydrous sodium acetate in a 1 : 2 molar ratio catalyst in 60 mL of acetic anhydride. The mixture was maintained under vigorous stirring in a water bath at 85°C and at reflux for 4 hours. The reaction was filtered under vacuum, followed by incubation under gentle agitation at 4°C. Then, 60 mL of acidified water (pH 3) was added, and the mixture was then incubated for 24 hours and then filtered, washed with doubly distilled water, and dried at 40°C. The 3,5-dimaleimylbenzoic acid product was obtained in 80% yield. The purity of the synthesized compounds was verified using thin layer chromatography, melting point measurement, infrared spectrometry, and nuclear magnetic resonance spectroscopy.

### 2.2. In Silico Analysis

Characterization of the local and global reactivity indexes of the derived maleic anhydride and quercetin was performed by Gaussian version 09 and AIM2000. For molecular structures and properties, analyses were performed using Gaussian version 09, MarvinView, and Structure Checker. Molecular modeling was carried out based on the method reported by Andrade et al. using the GaussView 5.0 computational package and Gaussian version 09 [18]. Briefly, the method used in the optimization was B3LYP, which is based on the density functional theory; then, the frequencies were calculated using the same level of theory to confirm that the conformation has been found at a minimum local energy [18]. The wave function was calculated using the optimized *Z* matrix of each molecule; the input file for each molecule was generated using the B3LYP method. The ionic structures were determined using the theoretical model UB3LYP/6-31G. All analyses were performed with the Gaussian package version 09. Then, the generated files provided the value of the energy for each of the structures to calculate the global reactivity indexes. Afterward, the charges of each atom in all the neutral molecules and the respective ions were calculated. Finally, the local reactivity indexes were calculated according to the formulae described. The electronic population for the calculation of Fukui functions was based on the formulation of the quantum theory of atoms in molecules.

### 2.3. Cell Culture and Treatment

Human cancer cell lines (HuH7 provided by Dr. Zentella Dehesa and HepG2 obtained from American Type Culture Collection, ATCC) were maintained in Dulbecco's Modified Eagle's Medium (DMEM; Gibco, 12800-017) containing 1% L-glutamine, 10% fetal bovine serum, 100 U/mL penicillin, and 100 *μ*g/mL streptomycin in 5% CO_2_ at 37°C. As a control, we used two human epithelial cell lines (HaCaT provided by Dr. Enrique Perez and THLE-3 obtained from ATCC) and primary hepatocytes of male rat Fischer-344 were isolated following the method described by Berry and Friend with modifications (1969). HaCaT cells were cultured under the same condition as that of cancer cells. THLE-3 cells were maintained in BEGM (BEGM Bullet kit; Lonza, C3170) on plates coated with type I collagen under the conditions recommended by ATCC. Cells were grown until they reached 70% confluence in specific medium supplemented with 10% FBS; then, the cells were starved for 12 hours with 2% FBS. Compounds were immediately added, and the cells were incubated for 12, 24, and 48 hours. The group without treatment was considered negative control (NC). We used an aqueous solution containing 0.2% dimethyl sulfoxide (DMSO) as the vehicle for the compounds. The optimal dose for quercetin (Q, Sigma-Aldrich, 32,782) was 50 mM, and that for 3′5′-dimaleamylbenzoic acid (C1) and 3′5′-dimaleimylbenzoic acid (C2) was 0.01 mM. The optimal dose of each compound was used for each of the combination treatments, with the compounds administered 30 minutes apart.

### 2.4. Cell Viability and Cell Cycle Assays

The effect of the treatments on the viability of cells was determined using the MTT (3-(4, 5-dimethylthiazol-2-yl)-2,5-diphenyl tetrazolium bromide, Thermo Fisher Scientific, M6494) assay. Briefly, ELISA plates with each treatment groups were washed with fresh culture medium and then incubated in fresh medium containing MTT (0.5 mg/mL) for 3 hours at 37°C. The MTT-containing medium was discarded, and the cells were incubated in DMSO to dissolve the formazan aggregates. The intensity of the product was read at 570 nm using an ELISA microplate reader. For cell cycle analysis by flow cytometry, the cells were washed with PBS and incubated at 37°C with 0.25% trypsin and inactivated by adding conditioned medium with 10% FBS. Subsequently, the cells were centrifuged at 1000*g* for 5 minutes, and the pellet was resuspended in 1x PBS. The suspension was centrifuged again under the same conditions, the supernatant was discarded, and the cell pellet was fixed with EtOH (−20°C) added dropwise with slow stirring. Subsequently, the samples were centrifuged, 1x PBS was added, and the cell pellet was dissociated by pipetting, and the mixture was centrifuged again. Finally, the supernatant was discarded, and the cell pellet was resuspended in the staining solution (176 *μ*L of PBS, 4 *μ*L of 10 mg/mL RNase and 20 *μ*L of 1 mg/*μ*L IP, 200 *μ*L per sample) for 40 minutes at 37°C. Then, the cell cycle distribution was analyzed using a FACSCalibur system.

### 2.5. Fluorescent Staining and TUNEL Assay

After treatment, the cells were fixed with 4% paraformaldehyde at room temperature (RT) under gentle agitation. Subsequently, the cells were washed with 1x PBS and permeabilized with 0.1% Triton X-100-PBS. The cells were washed again with 1x PBS and stained with the nuclear fluorescent dye Hoechst (H3570 350/461) 1 : 5000 in 1x PBS for 5 minutes in the dark. It was washed, and the second fluorescent label phalloidin (Thermo Fischer, A12379) was added at 1 : 1000 in 1x PBS. The cells were washed again, and mounted with Vectashield mounting media on conventional slides. The samples were stored in a humid chamber at 4°C for no more than 2 weeks. They were finally observed under a confocal microscope. For TUNEL assay, cells were fixed with 4% paraformaldehyde at RT covered with aluminum foil with gentle shaking for 1 hour and subsequently washed with 1x PBS and permeabilized with 0.1% Triton X-100 and 0.1% sodium citrate with shaking for 2 minutes at 4°C. Then, the cells were washed with 1x PBS, and the reaction was carried out in accordance with the instructions of the manufacturer (In Situ Cell Death Detection (fluorescein), Sigma-Aldrich; 1,684,795). The samples were then incubated at 37°C in complete darkness for 1 hour and then washed with 1x PBS and stained with Hoechst nuclear fluorescent dye (H3570 350/461) at 1 : 5000 in 1x PBS for 5 minutes. The cells were washed with 1x PBS and mounted with Vectashield mounting media on conventional slides and stored in a humid chamber at 4°C for no more than 2 weeks. Finally, they were observed under a confocal microscope.

### 2.6. Immunoblotting

After the respective treatments, the cells were washed two times with 1x PBS, scraped, and lysed in RIPA buffer containing a cocktail of protease inhibitors. The cell lysates were centrifuged for 10 minutes at 16000*g*. The supernatant was collected, and the protein content in the samples was determined using Bio-Rad protein assay reagent (Bio-Rad, 500-0113-14-15). The samples were mixed with 2x sample buffer (100 Mm TRIS-HCL pH 6.8, 4% SDS, 0.2% bromophenol blue, 5% *β*-mercaptoethanol, and 20% glycerol) and boiled at 95°C for 5 minutes. Proteins from the samples were resolved on 10–12% SDS-PAGE gels and then transferred to PVDF membranes for immunoblotting analysis. The membranes were blocked in 5% nonfat dried milk or 1% BSA in PBS-t (PBS-0.1% Tween 20) for one hour. Then, the membranes were incubated with the respective primary antibodies (caspase-9, sc-8355; caspase-8, sc-7890; and caspase-3, cell signaling #9662) overnight at 4°C. After that, the membranes were washed and incubated with HRP-conjugated secondary antibodies for two hours at RT and developed using chemiluminescent solution (Millipore, WBKLSO100).

### 2.7. Migration Assay

Cells were pretreated with 12 *μ*M mitomycin C to inhibit cell proliferation during the assay. Subsequently, a scratch was made on the cell layer with a 200 *μ*L micropipette tip. Cells were washed with 1x PBS, and DMEM without fetal bovine serum was added with the appropriate compounds corresponding to each of the treatment groups. The cells were incubated at 37°C with 5% CO_2_ for 24 and 48 hours, washed with 1x PBS, and fixed with 4% paraformaldehyde. Subsequently, the cells were stained with 0.5% violet crystal. The cells were then washed again with 1x PBS to remove excess dye.

### 2.8. Measurement of Reactive Oxygen Species and Glutathione Levels

The ROS assay was performed as described earlier (Chandel et al. [20]). Briefly, approximately 10,000 cells were seeded in 96-well plates in DMEM with 10% FBS and incubated at 37°C in 5% CO_2_ until they reached 80% confluence. The cells were then washed with Hank's saline solution (HBSS 1X) and cultured with 2% FBS for 12 hours. After serum starvation, they were again washed with 1X HBSS and conditioned medium with 2% SFB was added with the compounds corresponding to each of the study groups, and the cells were incubated for 24 hours. Subsequently, they were washed, and DCFDA working solution was added for 30 minutes at 37°C. Then, the fluorescence of DCFDA was measured using a Fluoroskan Ascent (Thermo Electron Corporation) fluorometer at ʎex: 480 nm and ʎem: 515 nm. The data were analyzed using GraphPad Prism. For the determination of glutathione concentration, cells were washed with Hank's saline solution and starved in DMEM with 2% FBS for 12 hours, followed by the corresponding treatments for 24 hours. Then, the cells were washed with Hank's saline solution. Trypsin was then then added for specific times for each type of cell line. Protein extraction from each of the experimental groups was then performed. The cell pellets were suspended in FEDTA (phosphate-buffered EDTA; 0.1 M monobasic sodium phosphate, 0.005 M EDTA pH 8.0) plus 25% phosphoric acid, followed by centrifugation at 16000*g* for 30 minutes. For the determination of reduced glutathione, 125 *μ*L of the above supernatant was taken and mixed with 1125 mL FEDTA, and 25 *μ*L was taken and mixed with 450 *μ*L of FEDTA and 25 *μ*L of O-phthaldialdehyde. For the determination of oxidized glutathione, 125 *μ*L of the above supernatant was taken and suspended in 50 *μ*L of 0.04 M N-ethylmaleimide and incubated for 30 minutes at RT. Subsequently, 1.07 mL of 0.1 N NaOH was added, and 25 *μ*L of the above mixture was added to it with 450 *μ*L of NaOH plus 25 *μ*L of O-phthaldialdehyde. This was mixed and left at RT for 15 minutes in complete darkness. The results were obtained using a fluorometer at ʎex: 350 nm and ʎem: 420 nm. The data were analyzed using GraphPad Prism.

### 2.9. Statistical Analysis

The data were expressed as the mean ± SEM for each analysis. Statistical analyses were performed by one-way ANOVA and Tukey's multiple comparison tests using GraphPad Prism 7.0 software. Values were considered significant when *P* < 0.05.

## 3. Results

### 3.1. In Silico Analysis and *In Vitro* Assay of the Compounds

After completion of the synthesis of maleic anhydride derivatives, they were identified using infrared spectroscopy and ^1^H nuclear magnetic resonance spectrometry, where the displacements exhibited a clear correspondence between the spectra and the composition and structure of the molecules, indicating a purity of 99%: C1, IR (ATR, cm-1) ύ: 3281.6 (NH), 1702.91 (C=O), 2800 (C-H, aromatic), C=C (1625.5) and ^1^H NMR (CDCl3, 400 MHz) *δ* 10.80 (s, H-NH), 8.30 (s, H-6), 7.97 (s, H-2′), 7.97 (s, H-4′), 6.32 (dd, H-2), 6.42 (dd, H-3), C2, IR (ATR, cm-1) ύ: 1722.8 (C=O), 3100 (C-H, aromatic), C=C (1600) and ^1^H NMR (CDCl3, 400 MHz) *δ* 7.66 (s, H-6), 7.99 (s, H-2′), 7.99 (s, H-4′), 7.59 (s, H-3), 7.59 (s, H-4).

**Figure 1:**
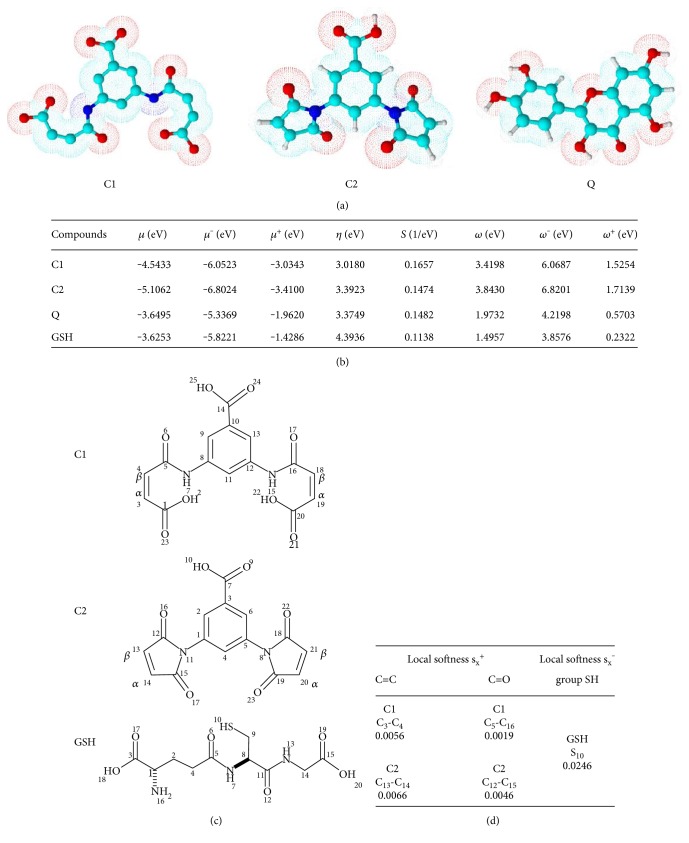
In silico analysis. (a) Geometric optimization of 3′5-dimaleamylbenzoic acid, C1, 3′5-dimaleimylbenzoic acid, C2, and quercetin, Q. (b) Global chemical and quantum reactivity descriptors: chemical potential, *μ*^−^ (eV); donor chemical potential, *μ*^−^ (eV); and acceptor potential, *μ*^+^ (eV); global hardness, *η* (eV); global softness (1/eV); electrophile index, *ω*(eV); electron-donating power, *ω^−^* (eV); and electron-accepting power, *ω^+^* (eV) under the same theory level B3LYP with the 6-31G basis. (c) IUPAC-based numerical assignment. (d) Local softness, s_x_^+^, of olefinic carbons and carbonyl carbon of C1 and C2 versus local softness, s_x_^−^, of the sulfur atom (S) of glutathione (GSH) assessed using Fukui condensed function.

In maleic anhydride derivatives, there are two places in each molecule that are susceptible to nucleophilic attack—the carbonyl carbon (by 1,2-addition) and the olefinic carbons (by Michael 1,4-addition), which are both electrophiles. However, the susceptibility of these carbons depends on their chemical softness or hardness. We carried out theoretical calculations in order to explore susceptibility, including global and local reactivity and consequent selectivity. In Figure 1(a), the geometric optimization is schematized under the same level of theory B3LYP/6-31G for compounds C1, C2, and Q. Chemical-quantum descriptors enable us to know the nucleophilic or electrophilic nature of a molecule globally or locally on a relative scale [96, 97 g]. The energies corresponding to the ionic structures (anion and cation) were calculated under the same level of theory using the UB3LYP/6-31G basis to prevent spin contamination (Figure 1(b)). The global parameters described are chemical potential (*μ* (eV)), donor potential *μ*^−^ (eV), acceptor potential *μ *^+^ (eV), global hardness *η* (eV), global softness S (1/eV), electrophilicity index *ω* (eV), electron-donating power *ω*^−^ (eV), and electron-accepting power *ω*^+^ (eV). GSH has a higher chemical potential, while the maleic anhydride derivatives have lower chemical potential. These results enabled us to predict that the electrons flow from GSH to *α*, *β*-unsaturated compounds, which is confirmed by the donor and acceptor potential where the electron flow occurs. For the case of Q, there was no representative difference in *μ* (eV) with respect to GSH. According to the hardness, *η*, interpretation, within the context of DFT, higher values of *η* indicate harder molecules, which are less reactive. GSH has a *η* value of 4.39, whereas C1, C2, and Q have values of 3.01, 3.39, and 3.37, respectively. Therefore, GSH is harder than the molecules studied. Likewise, the values of global softness, calculated as half of the reciprocal of hardness, show the same results as those of the hardness. In other words, lower softness corresponds to greater hardness. The results regarding the electrophilic index clearly show that the molecules C1, C2, and Q have values of 3.41, 3.84, and 1.97 s_x_^+^, respectively, maintaining an electrophilic behavior compared with the value of GSH. Electron-donating power and electron-accepting power are measurements of the ability of a chemical system to donate or accept a small fraction of the charge, respectively. The compounds C1, C2, and Q are acceptors, whereas the GSH molecule is a donor. The chemical structures for the compounds C1, C2, and Q are represented in Figure 1(c). In the local softness analysis, the olefinic carbons of C1 and C2 are more susceptible to thiol attack than the carbonyl carbons because they are highly electrophilic. On the other hand, the thiol group of GSH has a local softness, s_x_^−^, value of 0.024 at the sulfur atom (S) corresponding to the sulfhydryl group (SH), which has a nucleophilic behavior, so it can be an electron donor. Sulfur is the most suitable atom to carry out this attack against the olefinic carbons of C1 and C2, as it is a soft nucleophile (Figures 1(c) and 1(d)).

### 3.2. Synergistic Effects of the Combination of C1 and C2 with Q on Cell Viability

**Figure 2:**
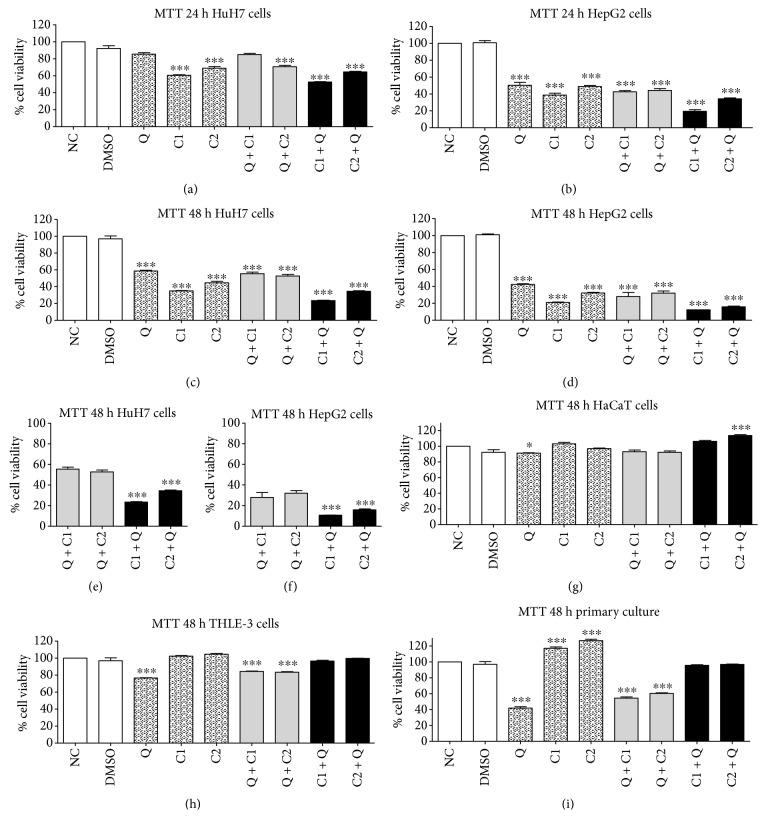
Effect on the viability of human liver cancer cells determined using MTT assay. (a), (c) HuH7 cells, (b), (d) HepG2 cells at 24 and 48 hours posttreatment. Effect on the viability of noncancerous human epithelial cells. (g) HaCaT cells, (h) THLE-3 cells, (i) primary culture of Fischer-344 rat hepatocytes at 48 hours posttreatment. (e), (f) Significant differences of the pleiotropic synergistic effect on cancer cells by Q + C1 and Q + C2 versus C1 + Q and C2 + Q. All data are presented as the mean ± SEM of 4 experiments; statistical evaluations were performed using Tukey's one-way ANOVA to obtain significant differences (^∗^*P* < 0.05, ^∗∗∗^*P* < 0.001) with normalization based on the vehicle group. NC, normal control; DMSO, vehicle; Q, quercetin; C1, 3′5-dimaleamylbenzoic acid; C2, 3′5-dimaleimylbenzoic acid.

One of the principal traits of cancer cells is their ability to sustain proliferation. The viability of cultured cells can be determined by the MTT assay. Metabolically active cells reduce the pale yellow tetrazolium salt (MTT) to purple-colored formazan. The absorbance of formazan correlates directly with the number of viable cells. Cytotoxic effects of Q, C1, and C2 were clearly observed in HuH7 and HepG2 at 12 hours after treatment (data not show). The results at 24 and 48 hours posttreatment indicated significantly higher toxicity in all the cancer cell lines tested. C1 had the strongest effect by itself with a 66.19% reduction in HuH7 cells and 80.2% in HepG2 cells at 48 hours (Figures 2(c) and 2(d)). The effect of the combination of Q with prooxidant compounds was not significantly different from the effect of each treatment alone. However, when the prooxidant compounds were administered prior to quercetin, the antiproliferative effect was significantly different (Figures 2(e) and 2(f)). The greatest effect was observed at 48 hours for the HepG2 cells. Interestingly, the C1 + Q treatment was the most effective combination exhibiting a reduction of 80.3% and 90.1% in the number of HuH7 and HepG2 cells, respectively, at 48 hours posttreatment (Figures 2(c) and 2(d)). The noncancerous human hepatocytes and epithelial cells, as well as the primary culture of hepatocytes from healthy rats, did not show significant changes at 12 and 24 hours posttreatment (data not shown). Subtle changes in HaCaT (Q and C2 + Q groups) and HepG2 (Q, Q + C1, and Q + C2 groups) cells were observed at 48 hours posttreatment (Figures 2(g) and 2(h)). The primary culture of hepatocytes was more sensitive to Q, Q + C1, and Q + C2 (Figure 2(i)). It is important to note that the most effective treatments (C1 + Q and C2 + Q) against the cancer cell lines did not have a significant toxic effect on noncancer cell lines in terms of compromised cell viability.

### 3.3. Synergistic Induction of S Phase Arrest during Cell Cycle Progression by the Combination of C1 and C2 with Q

**Figure 3:**
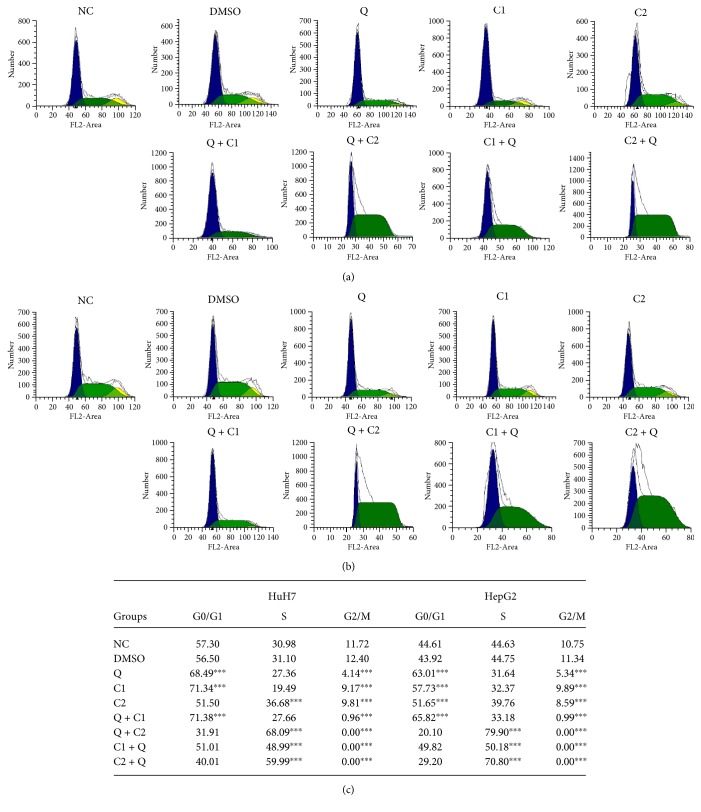
Cell cycle progression by flow cytometry in cells. (a) HuH7 and (b) HepG2 cells at 24 hours posttreatment; (c) treatment with Q, C1, C2, and Q + C1 arrests cells in the G0/G1 phase of the cell cycle, and treatment with Q + C2, C1 + Q, and C2 + Q arrests cells in the S phase of the cell cycle; the G2/M phase is affected by all treatments. All data are presented as the mean ± SEM of 4 experiments; statistical evaluations were performed using Tukey's one-way ANOVA to obtain significant differences (^∗∗∗^*P* < 0.001) with normalization based on the vehicle group treated with DMSO.

Cancer cells have the capability to continually respond to positively acting growth stimulatory signals. HuH7 and HepG2 cells were subjected to flow cytometric analyses following treatment. The results showed an S phase arrest following 24 hours of Q + C2, C1 + Q, and C2 + Q treatments in both cell lines (Figure 3). Quercetin by itself induced cell cycle arrest at the G0/G1 phase with 68.49% and 63.01% arrested HuH7 and HepG2 cells, respectively (Figure 3(b)). Additionally, C1 treatment induced G0/G1 phase arrest during cell cycle progression. The greatest effect was induced by treatment with C1 + Q, C1 + Q, and Q + C2 (*P* < 0.0001) with a 100% decrease in the fraction of cells in G2/M phase. Thus, the results show that the compound by themselves induce cell cycle arrest at G0/G1 phase, and in combination, the compounds arrest cells at S phase.

### 3.4. Effects of the Combination of C1 and C2 with Q on ROS Generation and Oxidative Stress

**Figure 4:**
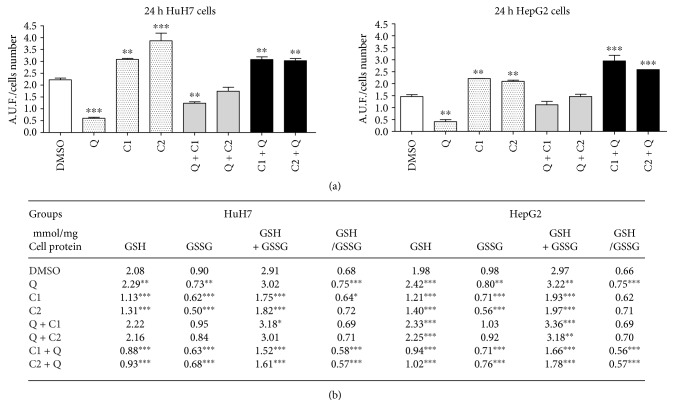
Effect of the treatments on ROS production assessed from the oxidation of DCFDA by hydrogen peroxide using fluorometric assay on HuH7 and HepG2 cells at 24 hours posttreatment; (b) determination of GSH and GSSG levels by fluorometric assay per mmol/mg protein at 24 hours posttreatment in HuH7 and HepG2 cells. All data are presented as the mean ± SEM of 3 experiments; statistical evaluations were performed using Tukey's one-way ANOVA to obtain significant differences (^∗^*P* < 0.05, ^∗∗^*P* < 0.01, and ^∗∗∗^*P* < 0.001) with normalization based on the vehicle group treated with DMSO.

To evaluate the possible cytotoxic effects of the combination of C1 and C2 with Q on the extent of oxidative stress, ROS generation and redox state of glutathione were determined by fluorometric analysis. Cancer cells subjected to the antioxidant treatment (Q) exhibited significantly reduced (*P* < 0.0001) ROS levels of 73.1% and 68.9% in HuH7 and HepG2 cells, respectively (Figure 4(a)). Treatment with Q + C1 and Q + C2 had a weak effect on decreasing the ROS levels in both cell lines. Treatment with the prooxidants (C1 and C2) increased ROS levels by 38.9% and 75.26%, respectively, in HuH7 cells. The compounds had a similar effect on HepG2 cells. Treatment with Q in combination with the prooxidant compounds tended to decrease ROS levels in both cell lines. The results show that the combination of the prooxidant compounds followed by quercetin increases the level of ROS. The antioxidant effect of quercetin was clearly demonstrated by the redox state of GSH, as there was a significant increase in the level of reduced glutathione and the GSH/GSSG index in addition to a decrease in the level of oxidized glutathione in both cell lines. Compounds C1 and C2 decreased the levels of reduced and oxidized glutathione by decreasing the GSH/GSSG index as well as the de novo synthesis of glutathione in both cell lines (Figure 4(b)). The application of quercetin followed by the oxidative compounds did not result in significant changes in the levels of reduced and oxidized glutathione in the HuH7 cells. However, the same treatments in the HepG2 line increased the level of reduced glutathione and the de novo synthesis of glutathione. In addition, when the prooxidant compounds were administered first, followed by treatment with quercetin, a significant decrease was observed in the levels of reduced and oxidized glutathione as well as in the de novo synthesis of glutathione and the GSH/GSSG index. These results demonstrate the modification of the redox state by prooxidant treatments and the effect of quercetin when it is administered before or after oxidative compounds.

### 3.5. Synergistic Effects of the Combination of C1 and C2 with Q in Cytoskeletal Actin and Nuclear Morphology

**Figure 5:**
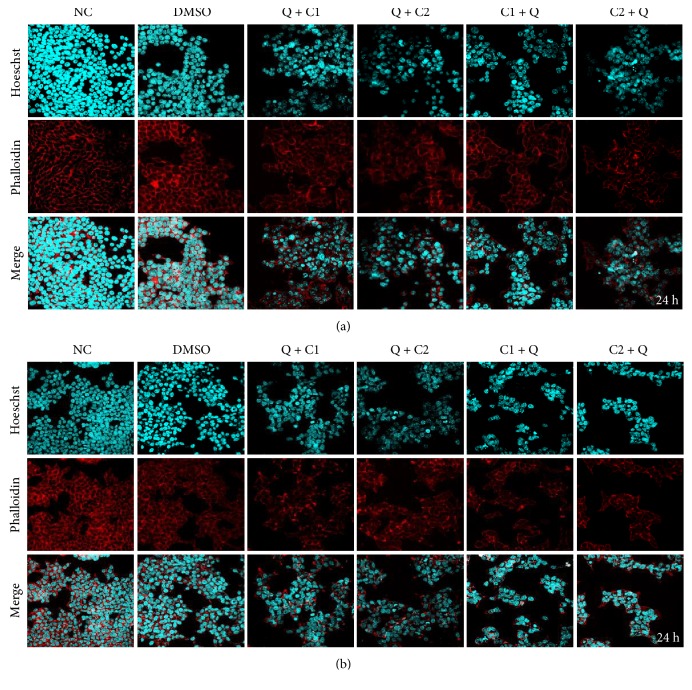
Synergistic effects of C1 and C2 in combination with Q on cytoskeletal actin and nuclear morphology in human liver cancer cells (a) HuH7 and (b) HepG2 at 24 hours posttreatment. Hoechst nuclear staining is shown in cyan, and F actin staining by phalloidin is shown in red at a magnification of 40x.

**Figure 6:**
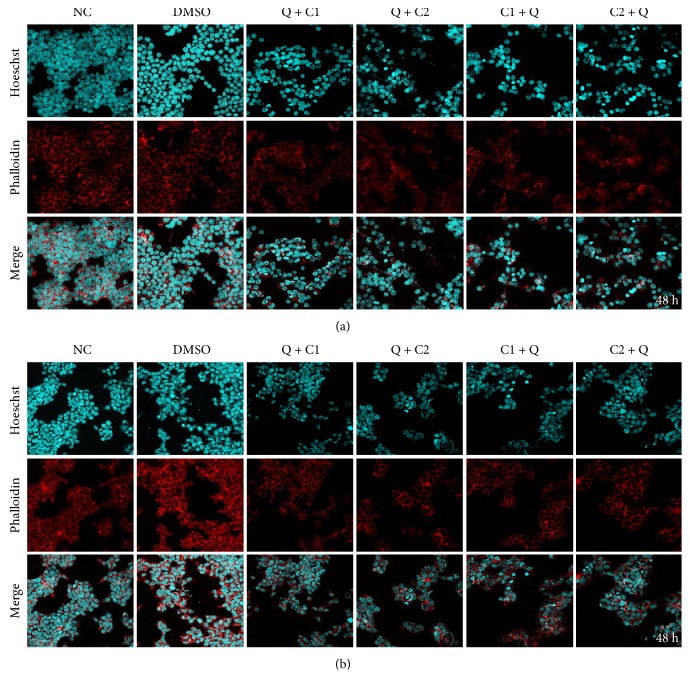
Synergistic effect of the combination of Q with C1 and C2 versus C1 and C2 with Q on cytoskeletal actin and nuclear morphology in (a) HuH7 and (b) HepG2 cells at 48 hours posttreatment. Hoechst nuclear staining is shown in cyan, and F actin staining by phalloidin is shown in red at a magnification of 40x.

Cancer cells are known to be exceptionally resistant to apoptosis. Hoechst H3570 is often used to distinguish condensed pyknotic nuclei in apoptotic cells, as it has the ability to easily cross the cell membrane due to its lipophilic nature. Actin was stained with phalloidin A12379 to observe if there were any changes. We observed nuclear condensation in all experimental groups at 24 hours posttreatment (Supplementary Material 1A and 1B available online at https://doi.org/10.1155/2017/2734976). The degradation of actin and DNA was observed mainly in the groups treated with combinations, and a stronger effect was observed on the HepG2 cells (Figure 5). At 48 hours posttreatment, we found more marked morphological change characteristic of apoptosis, with the treatment with C1 followed by quercetin being the most efficient (Figure 6). The apoptotic effect of the single administration of C1 was not significantly different in terms of toxicity in comparison to that observed when it was combined with quercetin (Supplementary Material 1). No effect was observed on cytoskeletal actin and nuclear morphology when quercetin was administered alone. The cell line HepG2 was the most susceptible to the apoptotic effects of the treatments.

### 3.6. Anticarcinogenesis Treatment Induces Apoptosis

**Figure 7:**
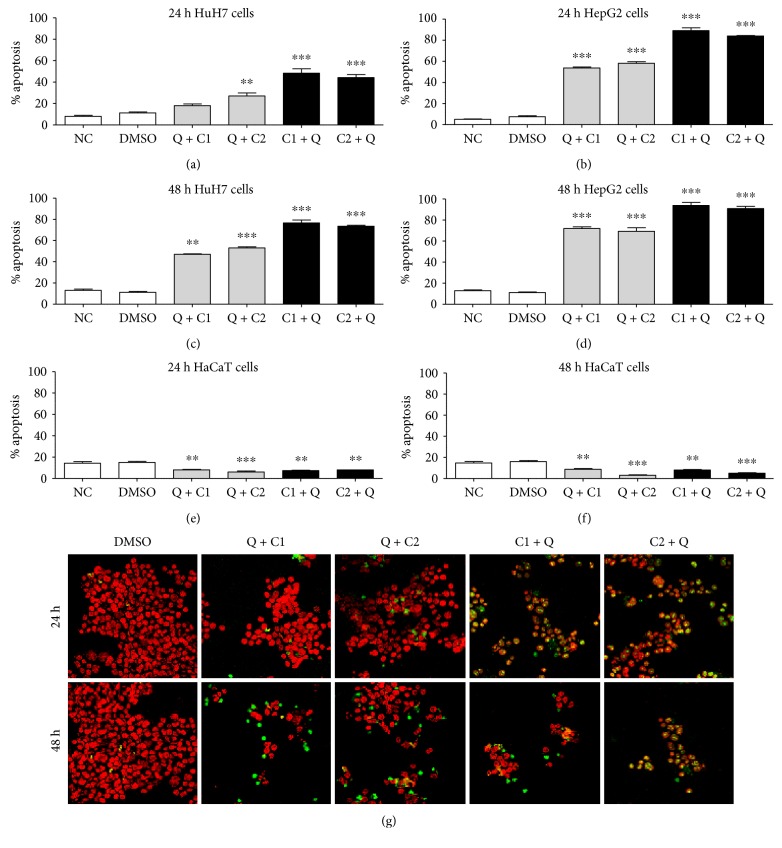
Quantification of pyknotic nuclei by staining with Hoechst in cells (a), (c) HuH7 cells, (b) (d) HepG2 cells, and (e), (f) HaCaT cells at 24 hours and 48 hours posttreatment. All data are presented as the mean ± SEM of 3 experiments; statistical evaluations were performed using Tukey's one-way ANOVA to obtain significant differences (^∗∗^*P* < 0.01, ^∗∗∗^*P* < 0.001) with normalization based on the vehicle group treated with DMSO. (g) TUNEL assay in HepG2 cells; nuclear staining of Hoechst is shown in red, and TUNEL is shown in green at a magnification of 40x.

The number of pyknotic nuclei (which correspond to fragmented DNA) were quantified at 24 and 48 hours posttreatment (Figures 7(a), 7(b), 7(c), 7(d), 7(e), and 7(f); Supplementary Materials 1E–H). There were statistically significant changes (*P* < 0.001) in most groups; the highest number of pyknotic nuclei was observed in the C1 + Q (48.3%) and C2 + Q (44.22%) treatment groups at 24 hours posttreatment, with an average of 75.1% in HuH7 cells at 48 hours posttreatment (Figures 7(a) and 7(c)). We observed the strongest effect in the HepG2 cells in response to C1 + Q (89.13) and C2 + Q (84%) at 24 hours posttreatment (Figures 7(b) and 7(d)). In general, single treatment with C1 was the most effective in inducing pyknotic nuclei with the maximum effect at 48 hours posttreatment (Supplementary Materials 1G and 1H). No significant changes were observed for each of the treatment groups at 12 and 24 hours in the noncancerous human cells that were stained with Hoechst and phalloidin. However, after prolonged exposure and starvation for more than 48 hours, some pyknotic nuclei were observed in the control cells. Figures 7(e) and 7(f) represent the quantified results for the apoptotic nuclei at 24 and 48 hours posttreatment, and the effect was minimal in the control groups. The number of cells positive for TUNEL (cells stained in green) was increased in response to treatment with C1 + Q and C2 + Q compared to those in response to the treatment with each of the compounds alone at 24 hours after treatment. This same behavior was observed at 48 hours posttreatment; however, the number of living cells was already lower at that time point than at 24 hours, which is why the number of fragmented nuclei was smaller. This assay demonstrated that the administration of the compounds individually leads to a lower extent of cell death.

### 3.7. Synergistic Effects of the Combination of C1 and C2 with Q on Activating the Intrinsic Pathway of Apoptosis

**Figure 8:**
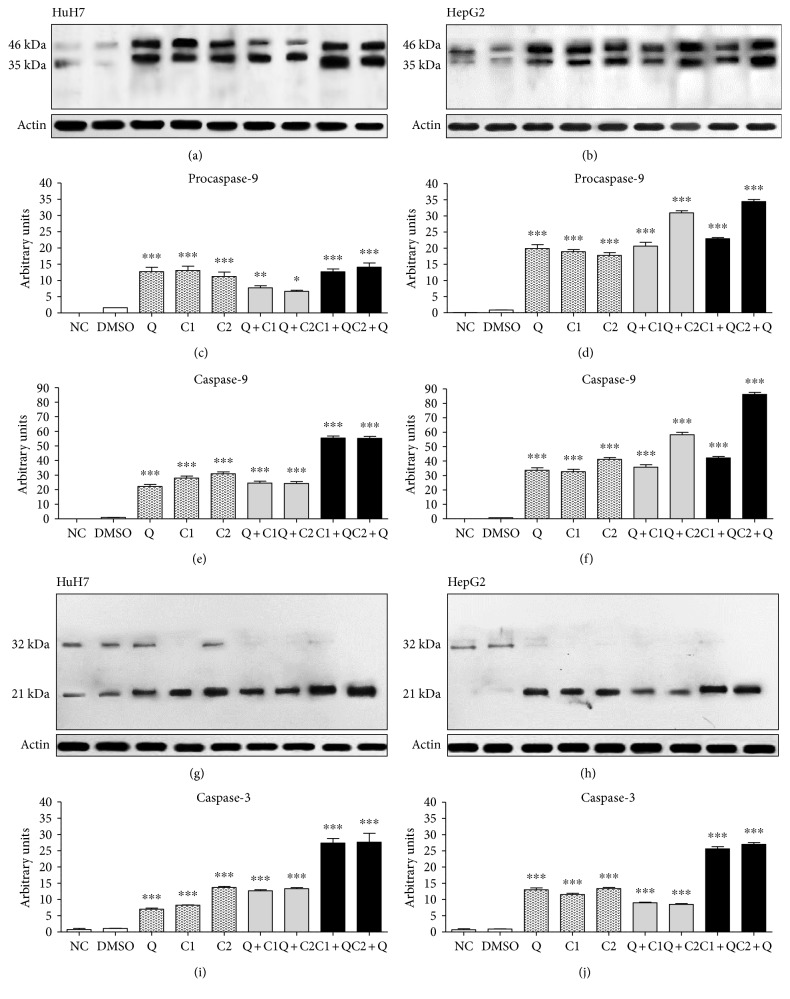
Activation of the intrinsic pathway of apoptosis after the effect induced by individual and combination treatments. Expression of procaspase-9 at 46 kDa and activation of caspase-9 at 35 kDa in (a) HuH7 and (b) HepG2 cells at 24 h posttreatment. Expression of procaspase-3 at 32 kDa and activation of caspase-3 at 21 kDa were observed at 36 hours posttreatment in (g) HuH7 and (h) HepG2 cells. For all cases, actin was used as a control of protein loading. (c), (d), (e), (f), (i), and (j) graphs corresponding to the colorimetric quantification by ImageJ. All data are presented as the mean ± SEM of 4 experiments; statistical evaluations were performed using one-way ANOVA with Tukey's test to obtain significant differences (^∗^*P* < 0.05, ^∗∗^*P* < 0.01, and ^∗∗∗^*P* < 0.001) with normalization based on the vehicle group treated with DMSO.

To determine the mechanism of apoptosis induced by the administration of C1 and C2 in combination with Q, Western blotting was performed. Cell lysates were prepared and analyzed for caspase-8, caspase-9, and caspase-3 expressions at 12, 24, 36, and 48 hours posttreatment. The results showed an increase in the level of procaspase-9 in all groups posttreatment (Figures 8(a), 8(b), 8(c), and 8(d)). The cleavage of caspase-9, an apoptotic marker, increased in all treatment groups, although the highest level was observed in the C1 + Q- and C2 + Q-treated HuH7 cells, and C2 + Q also strongly activated caspase-9 in HepG2 cells at 24 hours posttreatment (Figures 8(e) and 8(f)). The level of activated caspase-3 was confirmed in all treatment groups, although the highest level was observed in the C1 + Q and C2 + Q treatments in both cell lines at 36 h posttreatment (Figures 8(g), 8(h), 8(i), and 8(j)). The expression of procaspase-8 was observed from 12 hours onwards posttreatment in all experimental groups, and active caspase-8 was not observed (data not show).

## 4. Discussion

Hepatocellular carcinoma (HCC) is one of the most common causes of cancer-related death worldwide [21]. Similar to other types of cancers, HCC arises from a multistep and multifactorial process. Different risk factors determine the progression of HCC malignancy, and treatments are not efficient when it is detected. Although the relationship is not clear, diet has an important role in the development of HCC [22, 23]. This has led to the use of chemopreventive substances as alternative treatments. Antioxidants in the diet, such as flavonoids contained in several fruits and vegetables, have been used in animal models to show beneficial effects against liver tumors to induce apoptosis, and they have been shown to cause death in cancer cell lines [22, 24, 25]. Quercetin, a flavonoid widely studied as a chemopreventive agent in different types of cancer, is considered an excellent antioxidant [25]. It has been proven that quercetin inhibits metabolic activities and induces cell death by apoptosis in HCC cell lines such as HepG2, HuH7, and Hep3B2 [26]. Additionally, quercetin has been associated with the inhibition of enzymes that activate carcinogens and the suppression of key signal transduction pathways and receptor interactions. However, several studies have indicated that the anticancer activities and efficacy of quercetin can be further enhanced by combining it with other compounds [27–29]. The discovery of new drugs and novel therapeutic approaches for hepatocellular carcinoma opens the possibility of developing more effective strategies against various human cancers. In this regard, maleic anhydride derivatives have been shown to have anti-inflammatory and antiproliferative effects and to interfere with different cellular signaling pathways that depend on the availability of reduced thiols [30].

For the first time, our study demonstrates the cytotoxic effects of maleic anhydride derivatives (C1 and C2) on HuH7 and HepG2 cells (Figure 2). C1 and C2 can synergize with quercetin to inhibit cell viability. Additionally, we demonstrate the cancer preventive effect of quercetin and show that even when administered alone, C1 and C2 can exert cytotoxic effects on HuH7 and HepG2 cells at 24 and 48 hours after treatment. C1 and C2 were more cytotoxic against tumor cells compared to quercetin. The maximum of cytotoxic effect was observed when the compounds C1 and C2 were administered before quercetin. Interestingly, this effect was not observed in noncarcinogenic cell lines. These results suggest the selectivity of antitumor effect exerted by the compounds and indicate the possibility of a treatment approach that does not result in harmful effects on normal cells. However, the ability of quercetin to avert damage to normal cells has been previously reported [31]. The primary culture of hepatocytes was more sensitive to quercetin and the rest of the agents, except for the combinations C1 + Q and C2 + Q until 48 hours posttreatment. The noncancerous cells HaCaT and THLE-3 cells did not display drastic cytotoxic effects after 48 hours of treatment. Extensive studies have been conducted to determine the optimal antitumor dose of quercetin and other flavonoids, and the experimental results indicate that cell viability is inhibited by quercetin in a time- and dose-dependent manner [13, 32]. We observed that the combination of maleic anhydride derivatives followed by quercetin decreased cell viability with a specific and almost selective effect on tumor cells.

The transformation changes occurring during carcinogenesis include the ability to respond to growth factors and produce mitogenic signals [33]. The progression of the cell cycle implies a sequential activation of CDKs. To test the mechanism of antitumor effect in this respect, we analyzed cell cycle progression. Our data in HuH7 and HepG2 cells revealed that treatment with Q + C2, C1 + Q, and C2 + Q resulted in S phase arrest concomitantly with a reduction in the proportion of cells in the G2/M phase. Individual treatment with Q and C1 and combined treatment with Q + C1 induced arrest in the G0/G1 phase. A similar G0/G1 phase arrest by quercetin has been observed in HL-60, U937, and OE33 cells, resulting in caspase-dependent cell death [12, 34, 35]. These results demonstrate specific responses depending on the administration schedule of the compounds and the type of cells used. Quercetin induces cytotoxicity in cancer cell lines in a dose-dependent manner and activates the mitochondrial pathway of apoptosis [36]. In leukemic cell lines, quercetin induces S phase arrest during cell cycle progression in a dose-dependent manner, although Nalm6 cells exhibit maximum sensitivity to the cytotoxic effects of quercetin at relatively low doses (10 *μ*M). Breast cancer cell lines display limited sensitivity to quercetin; in T47D cells IC50 value was 160 *μ*M [36]. Quercetin was shown to induce cytotoxicity and lead to G2/M phase arrest in a dose-dependent manner in ovarian cancer cells. The G2/M phase arrest increased after treatment with 100 *μ*g/mL quercetin aglycone [37]. Interestingly, when quercetin was tested in ovarian cancer cells, the cells showed much less sensitivity, and at high doses of quercetin, the viability of normal ovarian cells was not significantly affected [32].

Cancer cells generate ROS due to their increased requirement for ATP; the imbalance between antioxidants and prooxidants results in oxidative stress that eventually promotes cell death [38]. However, due to deregulated redox balance, cancer cells escape programmed cell death regardless of the persistently higher ROS, in a more efficient manner than normal cells, while the higher intracellular levels of reduced glutathione promote cell survival in tumors. Additionally, anticancer drugs have been shown to exert apoptotic effects based on GSH depletion [39]. Our results showed that treatment of HuH7 and HepG2 cells with C1, C2, C1 + Q, and C2 + Q results in an increase in ROS levels and concomitant decrease in GSH. The combination of maleic anhydride derivatives and quercetin has a greater effect on the GSH/GSSH index. The *in vitro* analysis of maleic anhydride derivatives clearly showed a selective reaction with the thiol group of glutathione [18]. Our findings show that C1 and C2 decrease the levels of reduced glutathione in HuH7 and HepG2 cells. According to the results of our in silico analysis, maleic anhydride derivatives are electron acceptors and therefore have an electrophilic behavior. In addition, the oxidative effects of C1 and C2 are limited by quercetin. When quercetin is administered before the maleic anhydride derivatives, the depletion of ROS and reduced glutathione by C1 and C2 is restricted. However, the combination of maleic anhydride derivatives and quercetin resulted in a greater decrease in the level of reduced glutathione. Surprisingly, with this combination and order of administration, high ROS levels were observed despite the presence of quercetin. According to the results obtained, it is possible to conclude that changes in glutathione and ROS levels might account for the greater antitumor effect of the administration of C1 and C2 before quercetin. Quercetin and its potentially toxic oxidation products (semiquinone and quinone radicals) exert prooxidant effects within cells as a consequence of persistent exposure to persistent high ROS levels, and these radicals, with high reactivity toward thiols, react with GSH [24, 40, 41]. The other ways in which quercetin acts as a prooxidant may be by altering ROS metabolism due to the decrease in intracellular GSH or by downregulating heat shock protein (Hsp)-90 and inhibiting TRX reductase.

Since GSH is one of the main cellular free radical scavengers in addition to thioredoxin family members, a high glutathione index indicates redox balance and appropriate intracellular redox homeostasis. ROS are implicated in cell invasion and migration. Further, we show that antitumor compounds inhibit the migration of HuH7 and HepG2 cells (Supplementary 2A and 2B). It has been shown that quercetin can prevent cell migration and epithelial-mesenchymal transition by suppressing the expression of N-cadherin and vimentin in prostate cancer cell lines with no cytotoxic effect on normal prostate epithelial cells. The combination of antioxidants has shown potent and significant induction of apoptosis and suppression of cell proliferation, MMP secretion, cell invasion, cell migration, and angiogenesis. Similarly, quercetin has been shown to synergize with epigallocatechin gallate to inhibit stemness, invasion, and migration of prostate cancer cells [29].

Our results, therefore, show that concomitant effects of maleic anhydride derivatives and quercetin in HCC cell lines induced cytotoxicity by a deregulation in the adaptive stress responses (ROS increase and diminish reduced glutathione) reflected in cell cycle arrest at S phase. To confirm that the cytotoxicity effects induced by treatment with antitumor agents resulted in apoptosis, Hoechst staining and TUNEL assay were performed. Our data have validated the apoptotic effects of treatment with antitumor agents, with the highest effects with the administration of maleic anhydride derivatives before quercetin. Consistent with our findings, it has been previously reported that the decrease in intracellular-reduced glutathione and increase of reactive oxygen species trigger apoptosis. Additionally, several reports have demonstrated that increased ROS act upstream of caspase-3 activation. Accumulation of ROS after treatment with antitumor agents was shown to induce DNA damage and apoptosis by decreasing the mitochondrial membrane potential resulting in the release of cytochrome C [42]. To determine the mechanisms by which treatment with antitumor agents induce apoptosis, Western blotting was performed. The results showed an increase in the levels of procaspase-9 and caspase-9; however, no significant effects were observed in the activation of caspase-8. The observed increase in the level of activated caspase-3 and cleaved caspase-9 confirmed the activation of the intrinsic pathway of apoptosis. Our findings indicate that quercetin alone clearly decreases the reactive oxygen species and increases the levels of reduced glutathione, the GSH/GSSG index, and the de novo synthesis of glutathione, and despite this, it induces mitochondrial apoptosis. The effect of quercetin on HCC cells can be explained based on the previous studies that have attributed this effect to the direct interaction of quercetin with DNA, which enables it to modulate proapoptotic and antiapoptotic proteins, inhibit the PI3K/Akt pathway, and thus decrease survival.

## 5. Conclusions

The present study indicated that treatment with C1, C2, or Q individually exerts cytotoxic effect on tumor cell lines, but the combination of maleic anhydride derivatives and quercetin exacerbates the cytotoxic effects. HuH7 and HepG2 cell are highly sensitive to growth inhibition by treatment with C1 + Q and C2 + Q. The combination treatment can block cell cycle progression at the S phase, whereas the individual treatments inhibit the cell cycle at the G0/G1 phase. The cytotoxic treatment triggers the mitochondrial apoptotic pathway by regulating the expression of caspase-9 and activating caspase-3. C1 and C2 increased ROS levels, and quercetin depleted ROS production. The combination treatments C1 + Q and C2 + Q increased ROS levels and depleted GSH in HuH7 and HepG2 cells at 24 and 48 hours. These findings demonstrate the pleiotropic effects of the combination of maleic anhydride derivatives and quercetin on liver cancer cells and open the possibility of using their effective chemopreventive effects in hepatocellular carcinoma.

## Supplementary Material

Supplementary 1. Effect of individual administration of Q, C1 and C2 on cytoskeletal actin and nuclear morphology in human liver cancer cells. A ), B ) HuH7 cells and C), D) HepG2 cells at 24 hours and 48 hours post-treatment. Nuclear staining of Hoechst is shown in cyan and staining of the action F by phalloidin is shown in red at a magnification of 40X. Quantification of pycnotic nuclei by Hoechst staining in cells. E), G) HepG2 cells and F), H) HuH7 cells at 24 hours and 48 hours post-treatment. All the data presented have a mean ± SEM of 3 experiments; the evaluations are performed using Tukey՚s one-way ANOVA to obtain significant differences (∗ P <0.05, ∗∗ P <0.01, ∗∗∗ P <0.001) with normalization based on the vehicle group treated with DMSO. Supplementary 2. Wound closure assay. Effect on cell migration at 24 hours post-treatment in liver cancer cells A ) HuH7 and B ) HepG2; treatment with C1+Q and C2+Q resulted in an average inhibition of 43.45% with respect to the vehicle group. Normal control, NC; vehicle, DMSO; quercetin, Q; 3′5-dimaleamylbenzoic acid, C1; 3′5-Dimaleimylbenzoic acid, C2. Quantification performed with ImageJ.

## Abbreviations

Q: Quercetin
C1: 3′5′-dimaleamylbenzoic acid
C2: 3′5′-dimaleimylbenzoic acid
HCC: Hepatocellular carcinoma
ROS: Reactive oxygen species
GSH: L-c-glutamyl-L-cysteinyl-glycine
GSSG: Oxidized glutathione
DCFDA: 2′,7′-dichlorodihydrofluorescein diacetate
DFT: Density functional level of theory
MTT: 3-(4,5-dimethylthiazol-2-yl)-2,5-diphenyl tetrazolium bromide
DMEM: Dulbecco's Modified Eagle's medium
FBS: Fetal bovine serum
PI: Propidium iodide
CDKs: Cyclin-dependent kinases.
