# Supplementary figures and images for: Cytotoxicity, Oxidative Stress, Cell Cycle Arrest, and Mitochondrial Apoptosis after Combined Treatment of Hepatocarcinoma Cells with Maleic Anhydride Derivatives and Quercetin
Source: Oxid Med Cell Longev. 2017 Oct 10;2017:2734976. doi: 10.1155/2017/2734976 (PMC5661749; doi:10.1155/2017/2734976)

## Supplementary 1

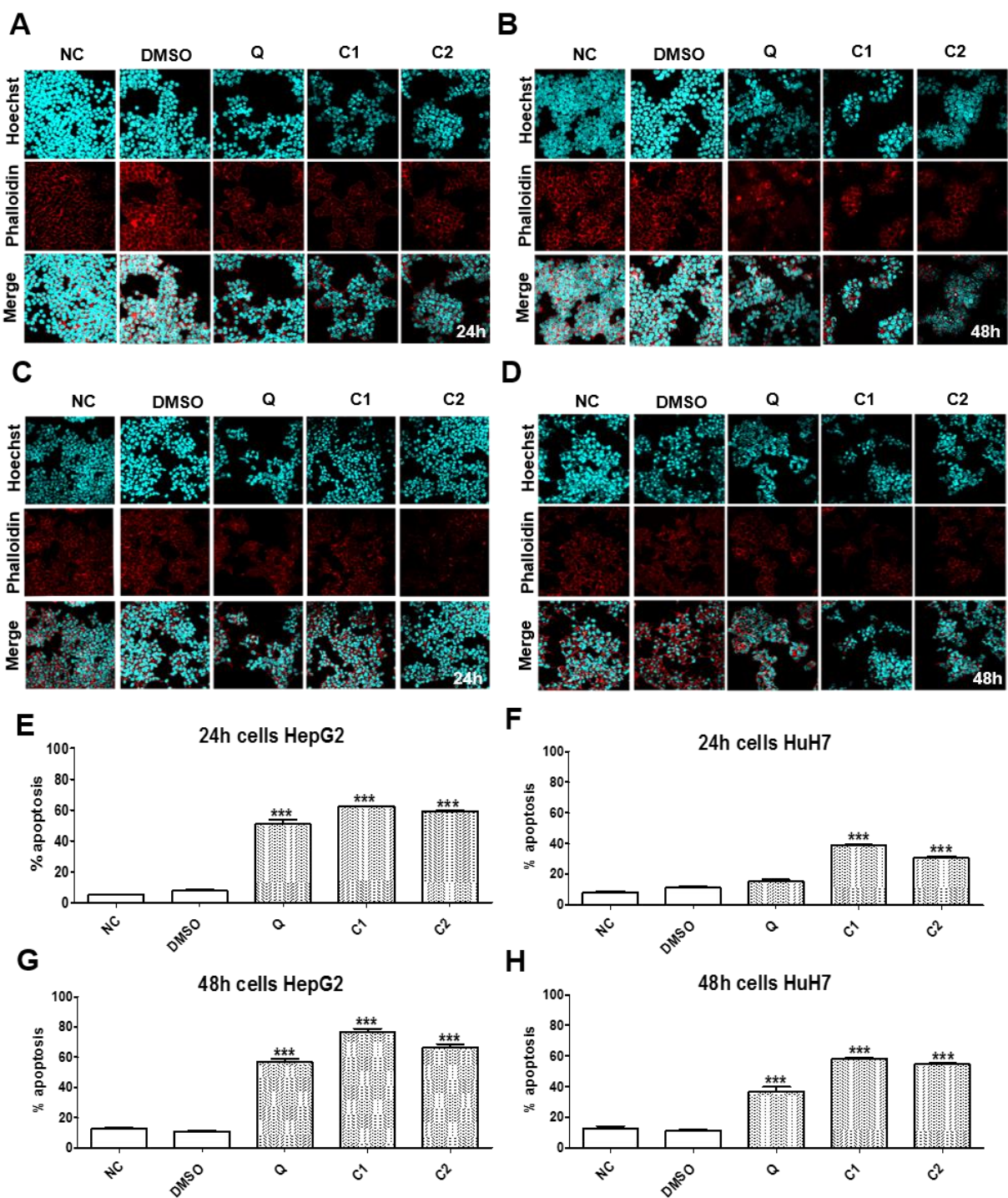

Supplementary 2

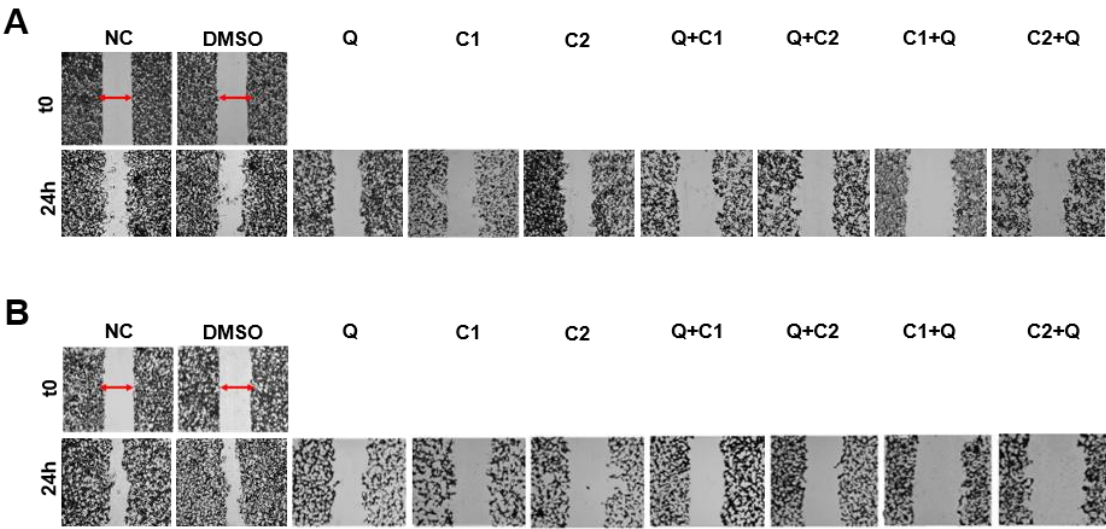

Supplement: Supplementary file 1 — Supplementary 1. Effect of individual administration of Q, C1 and C2 on cytoskeletal actin and nuclear morphology in human liver cancer cells. A ), B ) HuH7 cells and C), D) HepG2 cells at 24 hours and 48 hours post-treatment. Nuclear staining of Hoechst is shown in cyan and staining of the action F by phalloidin is shown in red at a magnification of 40X. Quantification of pycnotic nuclei by Hoechst staining in cells. E), G) HepG2 cells and F), H) HuH7 cells at 24 hours and 48 hours post-treatment. All the data presented have a mean ± SEM of 3 experiments; the evaluations are performed using Tukey՚s one-way ANOVA to obtain significant differences (∗ P <0.05, ∗∗ P <0.01, ∗∗∗ P <0.001) with normalization based on the vehicle group treated with DMSO. Supplementary 2. Wound closure assay. Effect on cell migration at 24 hours post-treatment in liver cancer cells A ) HuH7 and B ) HepG2; treatment with C1+Q and C2+Q resulted in an average inhibition of 43.45% with respect to the vehicle group. Normal control, NC; vehicle, DMSO; quercetin, Q; 3′5-dimaleamylbenzoic acid, C1; 3′5-Dimaleimylbenzoic acid, C2. Quantification performed with ImageJ. [file 2734976.f1.pdf]

## Slide 1
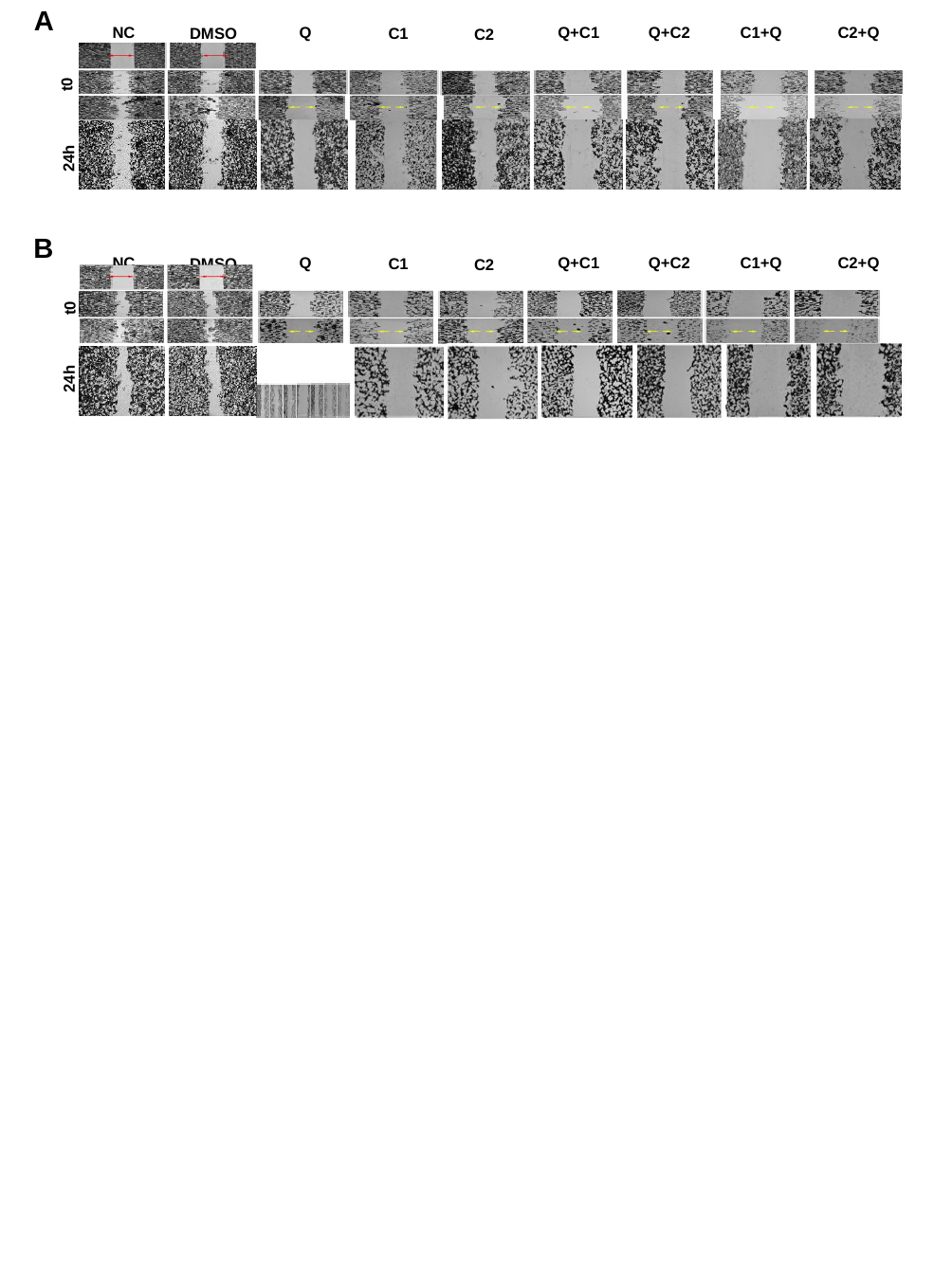

A
Q
Q+C1
Q+C2
C1+Q
C2+Q
NC
DMSO
C1
C2
t0
24h
B
Q
Q+C1
Q+C2
C1+Q
C2+Q
NC
DMSO
C1
C2
t0
24h

Supplement: Supplementary file 3 [file 2734976.f3.pptx]
