# Supplementary material for: Cytotoxicity, Oxidative Stress, Cell Cycle Arrest, and Mitochondrial Apoptosis after Combined Treatment of Hepatocarcinoma Cells with Maleic Anhydride Derivatives and Quercetin
Source: Oxid Med Cell Longev. 2017 Oct 10;2017:2734976. doi: 10.1155/2017/2734976 (PMC5661749; doi:10.1155/2017/2734976)
Supplement: Supplementary file 2 [file 2734976.f2.pptx]

## Slide 1
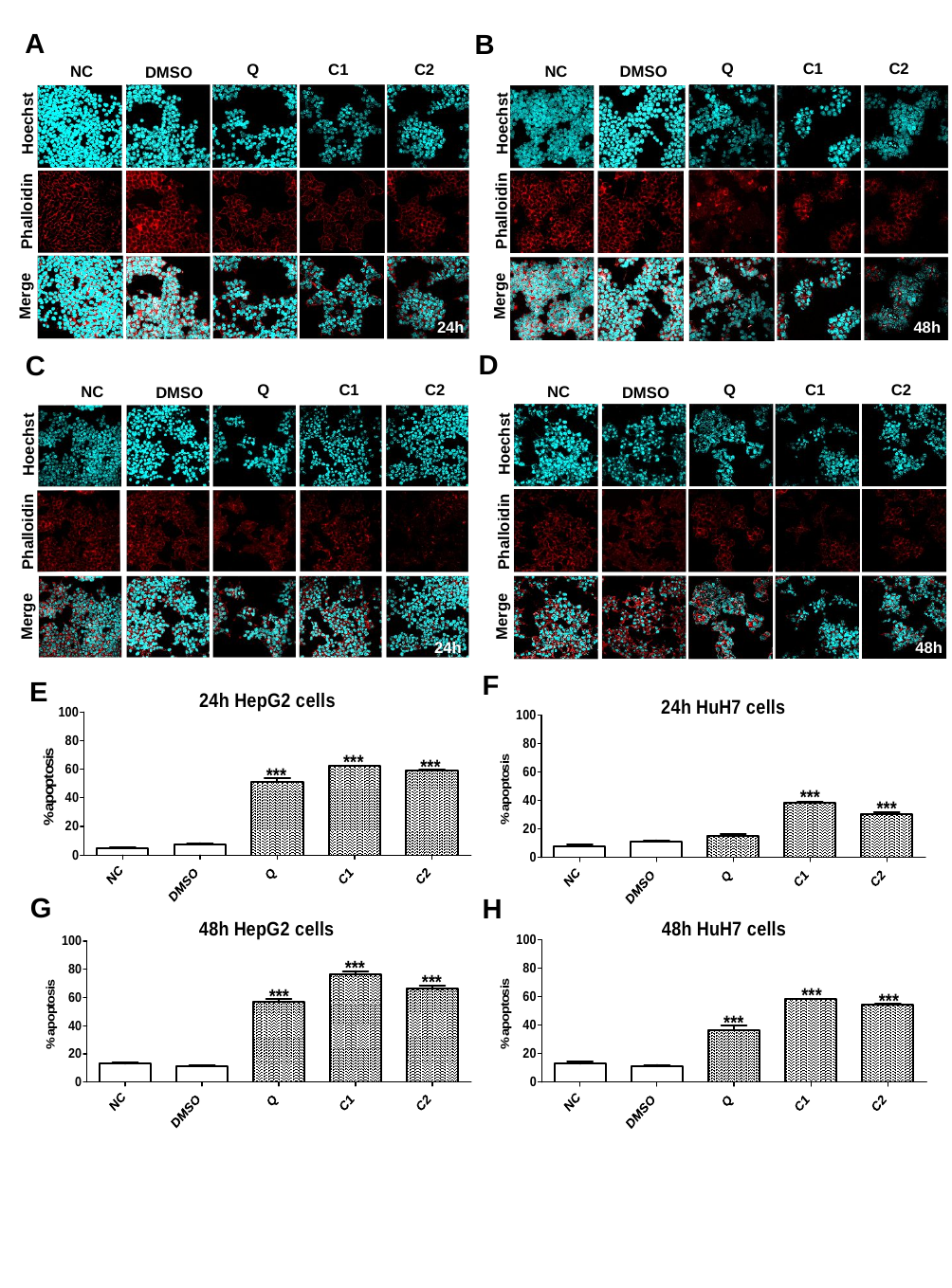

A
B
Q
C1
C2
Q
C1
C2
NC
NC
DMSO
DMSO
Hoechst
Hoechst
Phalloidin
Phalloidin
Merge
Merge
48h
24h
D
C
Q
C1
C2
Q
C1
C2
NC
NC
DMSO
DMSO
Hoechst
Hoechst
Phalloidin
Phalloidin
Merge
Merge
24h
48h
 F
 E
 G
 H
